# P-1183. Analysis of Cefiderocol use at an Academic Medical Center and Associated Patient Outcomes

**DOI:** 10.1093/ofid/ofaf695.1376

**Published:** 2026-01-11

**Authors:** Samantha Raush, Michelle Potter, Emir Kobic

**Affiliations:** Banner University Medical Center Phoenix, Phoenix, Arizona; Banner University Medical Center Phoenix, Phoenix, Arizona; Banner University Medical Center Phoenix, Phoenix, Arizona

## Abstract

**Background:**

Cefiderocol is a siderophore cephalosporin antibiotic approved for treatment of complicated urinary tract infections (cUTI), hospital-acquired bacterial pneumonia (HABP), and ventilator-associated bacterial pneumonia (VABP) caused by multidrug-resistant (MDR) gram-negative organisms. Banner University Medical Center – Phoenix (BUMCP) restricts cefiderocol use to specific MDR pathogens. Concerns exist about delayed initiation of cefiderocol due to limited rapid diagnostics, which may adversely affect patient outcomes.

**Table 1.**
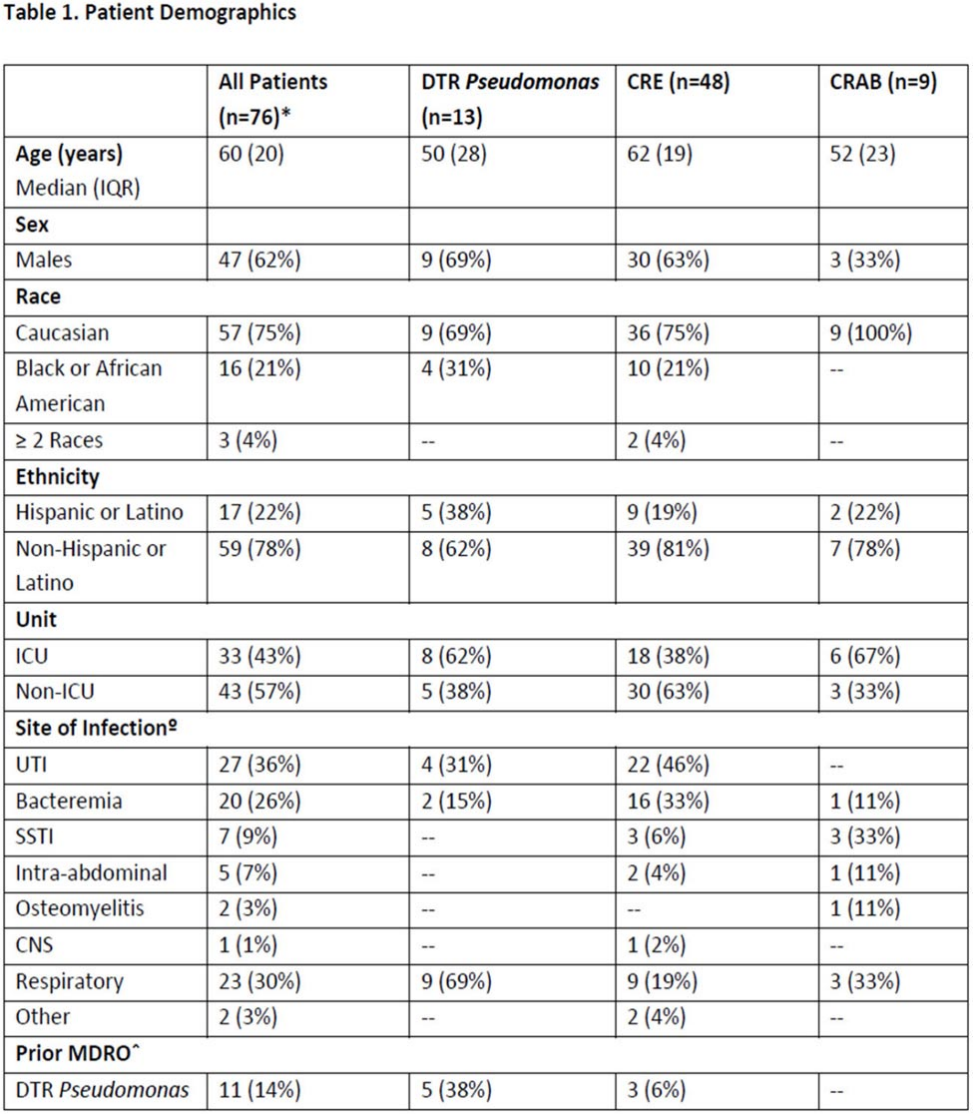
Patient Demographics

**Table 2.**
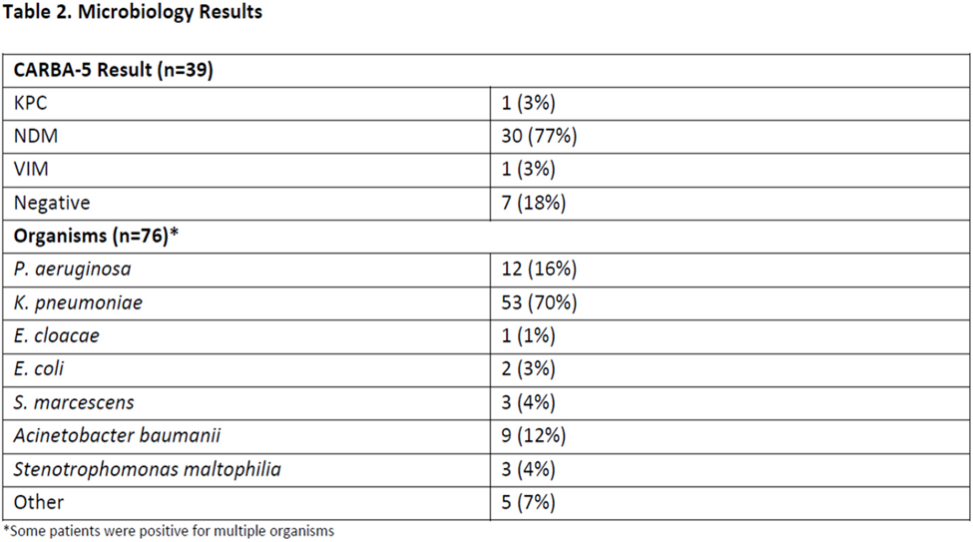
Microbiology Results *Some patients were positive for multiple organisms

**Methods:**

A retrospective chart review of hospitalized patients receiving ≥1 dose of cefiderocol from January 2023 to July 2024 at BUMCP was conducted. Patients were evaluated for clinical appropriateness of cefiderocol use, timing of initiation, microbiological susceptibilities, and outcomes including 30-day mortality, re-infection, and readmission.

**Table 3.**
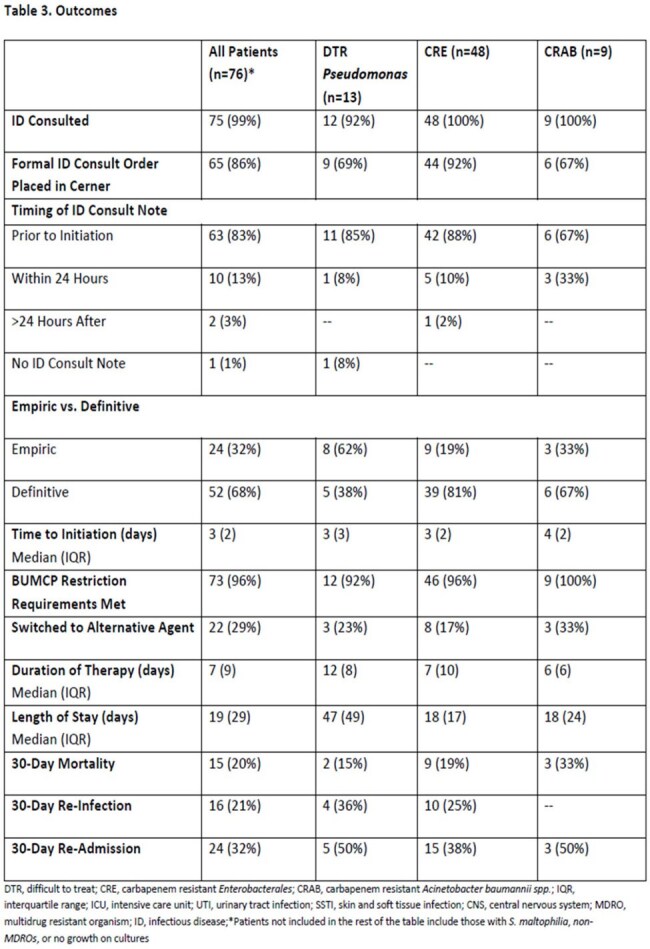
Outcomes DTR, difficult to treat; CRE, carbapenem resistant Enterobacterales; CRAB, carbapenem resistant Acinetobacter baumannii spp.; IQR, interquartile range; ICU, intensive care unit; UTI, urinary tract infection; SSTI, skin and soft tissue infection; CNS, central nervous system; MDRO, multidrug resistant organism; ID, infectious disease;*Patients not included in the rest of the table include those with S. maltophilia, non-MDROs, or no growth on cultures

**Results:**

Seventy-six patients were included. Cefiderocol was used primarily for CRE (63%) and CRAB (12%) infections. Most patients (96%) met BUMCP restriction criteria. Cefiderocol was used as definitive therapy in 68% of cases, with a median initiation time of 3 days post-culture. ID was consulted in 99% of cases. Thirty-day mortality was highest in CRAB infections (33%), consistent with CREDIBLE-CR trial findings. Susceptibility to cefiderocol was confirmed in 86% of organisms tested. Delays in susceptibility testing and inconsistent reporting of preferred agent susceptibilities were identified.

**Conclusion:**

Cefiderocol use at BUMCP is largely appropriate and reserved for high-risk MDR infections. Delayed initiation, often beyond the 48–72-hour window studied in clinical trials, may contribute to worse outcomes, particularly in CRAB infections. Implementation of routine CARBA-5 testing and reflex susceptibility panels may improve timely initiation. Further studies are needed to assess impact of diagnostic improvements on outcomes.

**Disclosures:**

Emir Kobic, BCIDP, Shionogi: Honoraria

